# Fostering Pro-environmental Behavior Among National Park Visitors: Testing Communication Strategies for Campfire Management

**DOI:** 10.1007/s00267-026-02392-6

**Published:** 2026-02-18

**Authors:** Sofie Selvaag, Marianne Evju, Vegard Gundersen, Øystein Aas

**Affiliations:** 1 Norwegian Institute for Nature Research (NINA), Høgskoleringen 9, Trondheim, Norway; 2 Norwegian University of Life Sciences (NMBU), Faculty of Environmental Sciences and Natural Resource Management, Høgskoleveien 12, Ås, Norway

**Keywords:** Environmental impacts, Visitor management, Recreation ecology, Information, Conservation

## Abstract

Effective communication is a key instrument for managing visitor impacts in natural areas. This study examines how passive (pamphlets) and active (face-to-face) communication strategies influence campfire behavior, with a focus on reducing the use of ancient trees for firewood. While campfires are a cherished activity worldwide, they can cause severe ecological damage. Using a multi-methods approach that integrates social and natural sciences, we conducted a quasi-experimental field study combining visitor interviews, surveys, observations, and environmental assessments. Our main analysis used a multiple regression model that reflects how communication operates under real-world conditions by accounting for visitor volume, visitor characteristics, and weather. Based on registered tree damage incidents, our results show that both communication types effectively reduced the number of tree damage incidents by 57% compared to days without communication. During communication periods, visitors more often refrained from making campfires or relied on provided firewood. The two communication strategies were similarly effective, challenging the common assumption in the literature that direct interaction works best. This suggests that simple, low-cost approaches to influencing visitor behavior can be powerful tools for environmental management, particularly in settings where staff capacity is limited.

## Introduction

Campfires in the wild are a long-standing cultural tradition and remain permitted also inside protected areas in many countries (Ryd 2005). Although managers often view fire pits and larger campfire sites as resource degradation, surveys indicate that visitors in some regions consider a single fire ring a desirable campsite amenity (Lucas 1980; Shelby et al. 1988). For many campers, campfires not only serve functional purposes such as cooking and drying clothes but are also vital for socializing and enhancing the camping atmosphere (Christensen and Cole 2000).

Campers often collect branches and logs of removable size from the surrounding forest areas for use in campfires, leading to loss of woody debris from the forest floor and branch material from standing living and dead trees (Hegetschweiler et al. 2009; Smith et al. 2012). Tree damage, including broken or cut limbs, hatchet wounds, and girdling, has an esthetic impact associated with campfires and has been found to have a negative effect on the quality of visitors’ experiences (Farrell et al. 2001; Lynn and Brown 2003). Importantly, woody debris play a vital role in forest ecosystems by variously contributing to soil processes and fertility, and hydrology, providing shelter for wildlife, serving as habitat for various invertebrates and birds, and offering substrates for plant germination and fungal growth (Seibold et al. 2015).

Various campfire management strategies have been developed and implemented to mitigate unwanted recreation-related resource impacts. Reid and Marion (2005) categorized such strategies into three types: *spatial*, which dictates where fires are allowed; *behavioral*, which promotes responsible visitor conduct such as using portable stoves, restricting the use of axes and saws, and controlling the sourcing of firewood; and *temporal*, which either specifies when fires can be lit, such as particular times of day or season, or allocates a set number of nights for campfires. Often, different types of management strategies are combined. For example, managers of outdoor recreation areas may only permit campfires in designated sites during seasons of low forest fire risk, while providing firewood for free use at the sites (Reid and Marion 2005).

Restrictive campfire policies, such as seasonal bans or confining fires to designated sites, have been widely implemented to minimize impacts on natural resources. However, such restrictions can conflict with wilderness ideals that emphasize freedom and self-reliance in interacting with the environment (Hammitt et al. 2015) and may undermine the quality of nature experiences or lead to visitor displacement (Gundersen et al. 2015). In many regions, direct legal restrictions face structural challenges due to rights of public access and freedom to roam (Fredman et al. 2021). However, Reid and Marion (2005) showed that a completely unregulated approach resulted in severe campfire-related damage and broader campsite impacts. More promising are strategies that modify practices without eliminating the activity. For example, the use of designated stoves has been shown to reduce impacts in U.S. wilderness areas where *leave no trace* principles are promoted (Christensen and Cole 2000), while providing firewood reduced tree damage in Australian outdoor recreation settings (Smith et al. 2012).

Building on these experiences, behavior-oriented approaches can reduce campfire impacts while still maintaining the quality of wilderness experiences. Communication is central, as it can shape visitor awareness and behavior in outdoor settings (D’Antonio et al. 2012). In this study, we tested targeted communication interventions in a Norwegian national park to identify effective ways of preventing the use of ancient trees for firewood. We tested the following hypotheses: (1) communication reduces tree damage, with lower levels of damage during periods when visitors receive information compared to control periods without communication, and (2) active communication, a short face-to-face message delivered by a national park employee, is more effective, i.e., reduces tree damage more than passive communication through pamphlets. By applying insights from communication and behavior-change theory, we also aimed to advance understanding of how such interventions operate under real-world conditions. Our study was designed to capture the complexity of natural settings, where ecological factors and situational dynamics cannot be fully reproduced in controlled experiments. The findings therefore highlight considerations that are critical for environmental management, particularly when developing communication strategies that balance recreational use with nature protection. Since communication is already widely applied as a management tool, it is essential to ensure that such strategies are effective.

## Literature Review

### Behavioral Changes Through Communication

All the campfire studies mentioned in the introduction emphasize the importance of environmental communication to change visitors’ behavior and inform them about alternative practices to improve campfire conduct (Christensen and Cole 2000; Reid and Marion 2005; Smith et al. 2012). *Unskilled actions*, such as improperly building a campfire or insufficiently putting out the fire, and *uninformed actions*, such as cutting trees for firewood without realizing the consequences, can have significant environmental repercussions (Marion and Reid 2007). To our knowledge, subsequent practice and guidance have continued to reflect these conclusions, and the underlying mechanisms and recommended actions remain largely unchanged. Environmental communication can play a vital role in addressing these impacts by aiming to provide visitors with the necessary knowledge and skills to minimize their impacts (D’Antonio et al. 2012). Communicating responsible campfire behavior has proven difficult because regulations and messages from land managers are often inconsistent (Aas et al. 2022; Stubbs 1991), and because campfire practices are frequently culturally rooted (Ryd 2005) or shaped by habit and routine, making them particularly resistant to change when they are accepted traditions elsewhere or have long been part of outdoor recreation (Stern 2000). For example, Serenari et al. (2012) found that in the Indian Himalaya, where making campfires was an accepted cultural behavior, rafting and hiking guides were reluctant to refrain from cutting living trees for firewood in the absence of any social pressure to do so. Campfire behavior can therefore be seen as hard to change due to social norms and established practices. However, He et al. (2023) highlight the need to prioritize studies of communication addressing behaviors that can be hard to change but that have significant environmental impacts.

Research indicates that nature-based tourists generally value educational experiences and are receptive to information about environmental issues (Ardoin et al. 2015). Communication can not only spark interest in biology, ecology, and conservation practices (D’Antonio et al. 2012) but also enhance visitor engagement in pro-environmental behavior while enriching their overall experience. Visitors often value site-specific information on how local ecosystems function and the species they may encounter (Marion and Reid 2007). When the rationale for rules is explicit, visitors are more receptive to guidance, consistent with Marion and Reid’s finding that messages work better when they explain the reasons behind site regulations. Relevant, engaging, and entertaining communication that includes diverse viewpoints can deepen emotional connection and foster stewardship (Stern and Powell 2013). Clear, well-designed messages have been shown to be effective in promoting responsible outdoor recreation practices (Ardoin et al. 2015; He et al. 2023). Both *passive* communication (e.g., signs and brochures) which relies on visitors voluntarily engaging with written material and *active* communication (e.g., on-site ranger information), which involves direct personal interaction and the possibility for dialog, have been focal points of study related to the sustainable management of protected and recreational areas (Kidd et al. 2019; Selvaag et al. 2023). Face-to-face communication is often more effective because it opens for natural conversation and for adjusting messages to visitors’ needs, while also using non-verbal cues like gestures and tone of voice (Keller et al. 2025). Nevertheless, most studies on communication in outdoor recreation have primarily investigated written, passive messaging (Selvaag et al. 2023), and there are conflicting findings regarding the superiority of personal contacts over brochures for reaching visitors (Oliver et al. 1985; Roggenbuck and Berrier 1981). To our knowledge, no comparable field experiments have revisited this question since the 1980s. To address this gap, we compared a brief face-to-face message with a pamphlet in a national park setting.

### Theoretical and Methodological Perspectives

A recent systematic review (Esfandiar et al. 2022) emphasizes the importance of focusing on specific visitor behaviors within particular contexts and measuring actual behavior through experimental study designs in protected areas. Similarly, He et al. (2023) make the same recommendation in the field of environmental communication in nature-based tourism and highlight the value of systematically comparing delivery mediums. Selvaag et al. (2023), in their review of outdoor recreation research, note that much existing work has focused primarily on written messages and North American contexts. Building on those insights, our study broadens the perspective by examining the concrete behavior of campfire practices in a European protected area. Norwegian national parks are primarily designated for conservation and often align more with IUCN Category Ib, wilderness area, than Category II, national park, including large, remote areas, simple infrastructure, and relatively low visitor densities (Gundersen et al. 2015). In Norway and other Nordic countries, *freedom to roam* (*allemannsretten*) guarantees public access to uncultivated land regardless of ownership or protection status, so indirect conservation mitigation measures, such as communication, become primary visitor-management tools (Fredman et al. 2021).

We applied an experimental field methodology, combining quantitative surveys and direct observation, to test the effectiveness of different communication approaches. Importantly, we compared not only written messages but also face-to-face communication. In doing so, we measured actual behavioral changes and their associated environmental impacts. This is particularly important given the well-documented inconsistencies between self-reported behavior or behavioral intentions and observed behavior (He et al. 2023; Schwartz et al. 2018). Furthermore, behavioral changes, whether intentional or unintentional, can have significant and sometimes unforeseen consequences for the environment (e.g. Marion et al. 2008; Solvason 2015).

Two major lines of theory dominate research on communication for influencing visitor behavior in nature-based tourism and outdoor recreation (Esfandiar et al. 2022; Selvaag et al. 2023). The first can be described as *behavioral theory*, which emphasizes rational and moral processes shaped by values, attitudes, beliefs, and norms. Within this line, the Theory of Planned Behavior (TPB) (Ajzen and Fishbein 1980) has been widely applied to explain how attitudes, social norms and perceived control influence intentions and behavior (Esfandiar et al. 2022). An important extension of this approach is found in norm-based theories, which highlight how environmental behaviors are motivated by individuals’ internalized moral obligations. The Value-Belief-Norm (VBN) theory (Stern 2000) underscores the role of personal norms, suggesting that individuals are more likely to engage in pro-environmental behavior if they believe their actions can prevent environmental harm and if they feel personally responsible for the outcomes.

The second line is *communication theory*, which focuses on the design and delivery of persuasive messages and how individuals interpret them depending on the source, message, receiver, and context. This perspective includes models of information processing such as the Elaboration Likelihood Model (Petty and Cacioppo, 1986). These theories highlight that the effectiveness of communication depends not only on content, but also on delivery, credibility, and timing. Importantly, these two theoretical lines are not mutually exclusive but instead complement each other. Behavioral theories explain the underlying psychological and normative predispositions that shape visitors’ willingness to act pro-environmentally, while communication theories clarify how message design and delivery can activate, reinforce, or reshape those predispositions. Michie et al.’s (2014) COM-B model of behavior change provides an integrative framework that helps bring these perspectives together in practical applications (Michie et al. 2014). This framework addresses both the motivations behind visitor actions and the external factors that can either facilitate or hinder those behaviors, while also guiding the selection of appropriate interventions and strategies that are specific to the place and behavior in question. According to this model behavior is shaped by; Capability: enhancing knowledge, skills, and psychological capacity to perform desired behaviors, Opportunity: ensuring that external factors, resources, and infrastructure support behavior, and Motivation: influencing attitudes, beliefs, and perceptions to encourage positive actions. The model emphasizes that visitors’ capability, opportunity, and motivation must be addressed simultaneously in order to drive meaningful behavioral change. By applying such integrative models, researchers and managers can design communication strategies that both resonate with visitors’ values and norms and optimize how messages are delivered and interpreted. In this study, we combine these perspectives by considering decision-making processes, moral and normative reasoning, and the role of communication stimuli in shaping visitor responses. Concretely, we conducted a theory-informed pre-study (TPB, VBN) to identify capability, opportunity, and motivation barriers to low-impact campfire practices, translated those barriers into message content and delivery choices aligned with COM-B, implemented a study with three communication conditions (control, passive, and active,) and evaluated effects by linking social data to ecological data and outcomes. Such coupled approaches remain scarce, yet they are crucial for understanding visitor behavior and environmental outcomes, given the intertwined nature of social and ecological drivers and effects (Monz et al. 2010).

## Materials and Methods

A qualitative pre-study with visitors (semi-structured interviews) in Femundsmarka, Norway, was conducted in 2022. The interview guide drew on the Theory of Planned Behavior and the Value-Belief-Norm framework, and findings were organized within the COM-B model to identify which components of campfire behavior to target (see Online Resource 2). Based on this, we designed the message content and chose delivery medium, timing, and location. In summer 2023, we implemented a quasi-experimental field study to test two hypotheses: **H1**, that communication reduces tree damage relative to no communication, and **H2**, that active communication is more effective than passive. The study used three sequential periods: (i) control (no message), (ii) passive (pamphlet distributed on the ferry), and (iii) active (one-minute ranger talk at disembarkation – see below). We mapped tree damage (environmental survey) as the primary outcome and modeled damage per site/day as a function of communication condition, controlling for visitor volume (manual counts of people at each campsite), visitor attributes (questionnaires), and weather conditions (Yr 2023). H1 was tested by comparing the pooled communication periods against the control; H2 by comparing the active period with the passive period.

### Study Area

Femundsmarka National Park (hereafter referred to as Femundsmarka), in southeast Norway (at approximately 62^o^1’ – 62^o^5’N, 11^o^6’ – 12^o^3’E), was protected in 1971 to preserve a large wilderness-like area of diverse forests, mountains, lakes, and rivers that contained extensive nature diversity. The studied area is dominated by old natural pine forest, including ancient trees and logs, and hosts a variety of insects, fungi and lichens. It is documented that since the early 1990s the level of tree damage from recreation has increased significantly (Aas et al. 2022). The area is remote but nevertheless popular in the summer months from June to August, when it attracts multiple-day visitors who engage in activities such as canoeing, fishing, and hiking (Vorkinn 2016). Given the limited availability of cabins, most visitors opt for tent accommodation and rely on campfires for various purposes, such as drying clothes and staying warm. Many visitors are drawn to the park to experience wilderness, remoteness, embark on extended hikes with minimal encounters with others, and enjoy the simplicity of few amenities (Vorkinn 2016). The area is managed by a local National Park Board in partnership with the Norwegian Environment Agency, with on-the-ground work, monitoring and enforcement carried out by park staff/rangers who conduct routine patrols and provide visitor information. This dynamic presents a compelling case for investigating how environmental communication and management measures can address visitor behaviors while maintaining the park’s character of solitude and freedom.

**Fig. 1:**
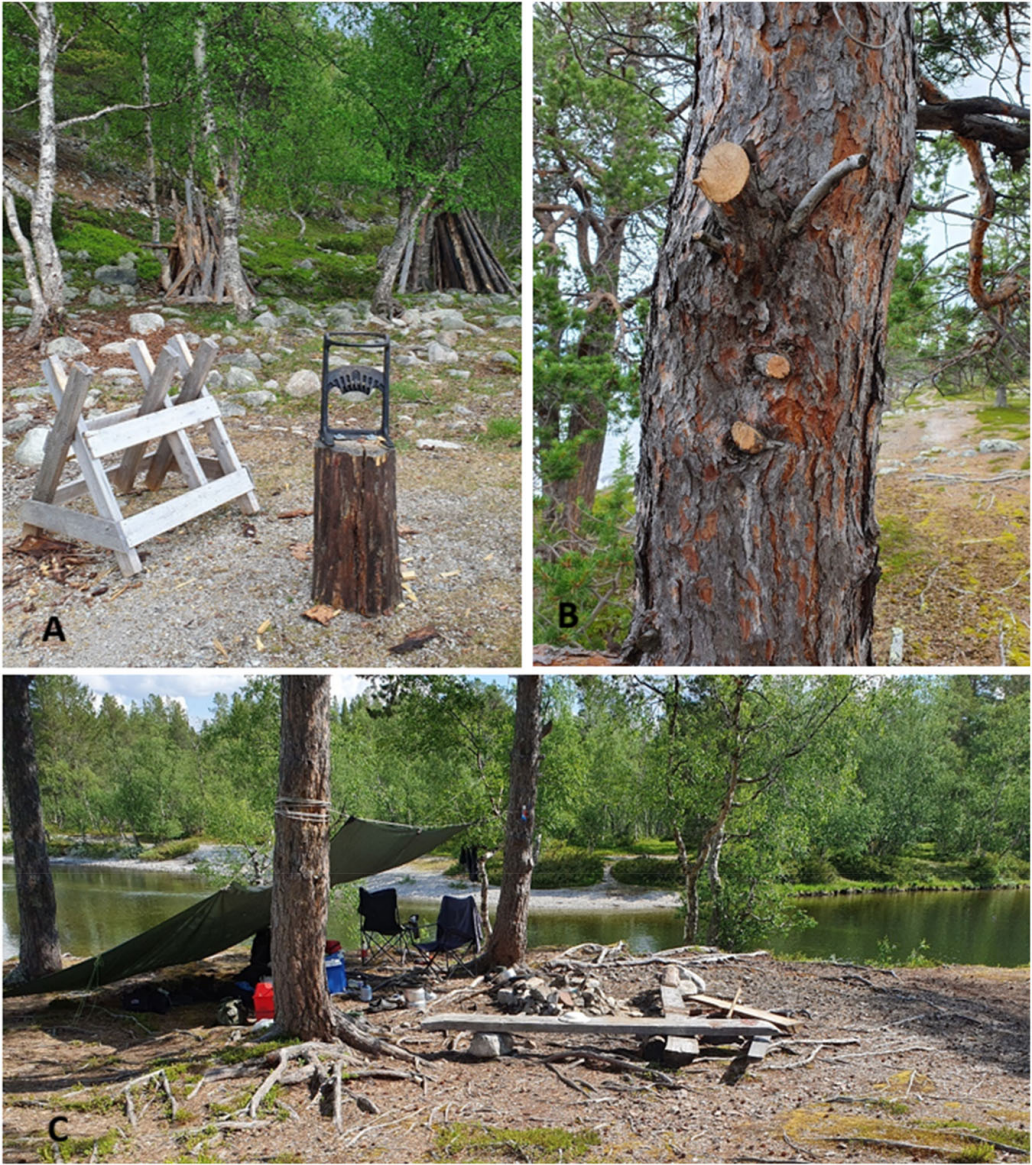
Typical campsites in the Røsanden area of Femundsmarka National Park and evidence of typical campfire behavior. **A** Provided firewood and tools for cutting and splitting wood, **B** an example of severe and illegal tree damage with cut branches from ‘*campsite 1*’. **C**) ‘*campsite 4*’ with tree damage incidents (campsites and provided firewood locations detailed in Fig. 3)

Our study area, Røsanden, in northwest Femundsmarka, is the most used entry point to the national park and almost all visitors are transported by the Fæmund II ferry, which runs daily to Røsanden during summer (Vorkinn 2016). Røsanden features several designated campsites equipped with wooden benches, fire rings (Fig. 1), as well as a composting toilet. In recent years, visitor management has intensified in an attempt to reduce the negative impacts of tourism and has included efforts to change visitor behavior to reduce tree damage caused when collecting firewood. Despite legal regulations stipulating the use of only small twigs, preferably from the ground, for campfires, it is a cultural tradition in the area to use dry wood (logs, stumps and branches) (Aas et al. 2022). This practice, which is deeply ingrained in Norwegian outdoor traditions, is particularly prevalent at Røsanden (Aas et al. 2022). To prevent depletion of wood resources, the park management provides firewood and tools for cutting and splitting near popular campsites (Fig. 1). The park management places a strong emphasis on communication to achieve the overarching goal of preserving the park’s natural character, including its ancient pines, while encouraging more sustainable visitor behavior (Ødegaard et al. 2021).

### **Designing the** Field Experiment

The multi-methodology used in our study consisted of four main components that also were combined analytically: (1) mapping tree damage (environmental survey), (2) documenting visitor use (manual counts, questionnaire survey), (3) understanding campfire behavior (questionnaire survey), and (4) mapping how communication influenced campfire behavior (environmental survey, observations). The study was carried out in the summer 2023, following a pre-study conducted in 2022 to understand campfire behavior and craft interventions in accordance with the COM-B model (qualitative interviews held with visitors; see Online Resource 2 for details). Fieldwork in 2022 was also used to plan, pretest, and design the 2023 quasi-experimental study conducted in the field (Campbell and Stanley 2015). Regular workshops and meetings were held with local stakeholders and the national park management to design management measures that encourage long-term solutions and high levels of “ownership” (Brooks and Champ 2006; Measham and Lumbasi 2013). No sensitive or personally identifying information was collected from participants in this study and the project received approval from the Norwegian Agency for Shared Services in Education and Research (SIKT).

**Fig. 2:**
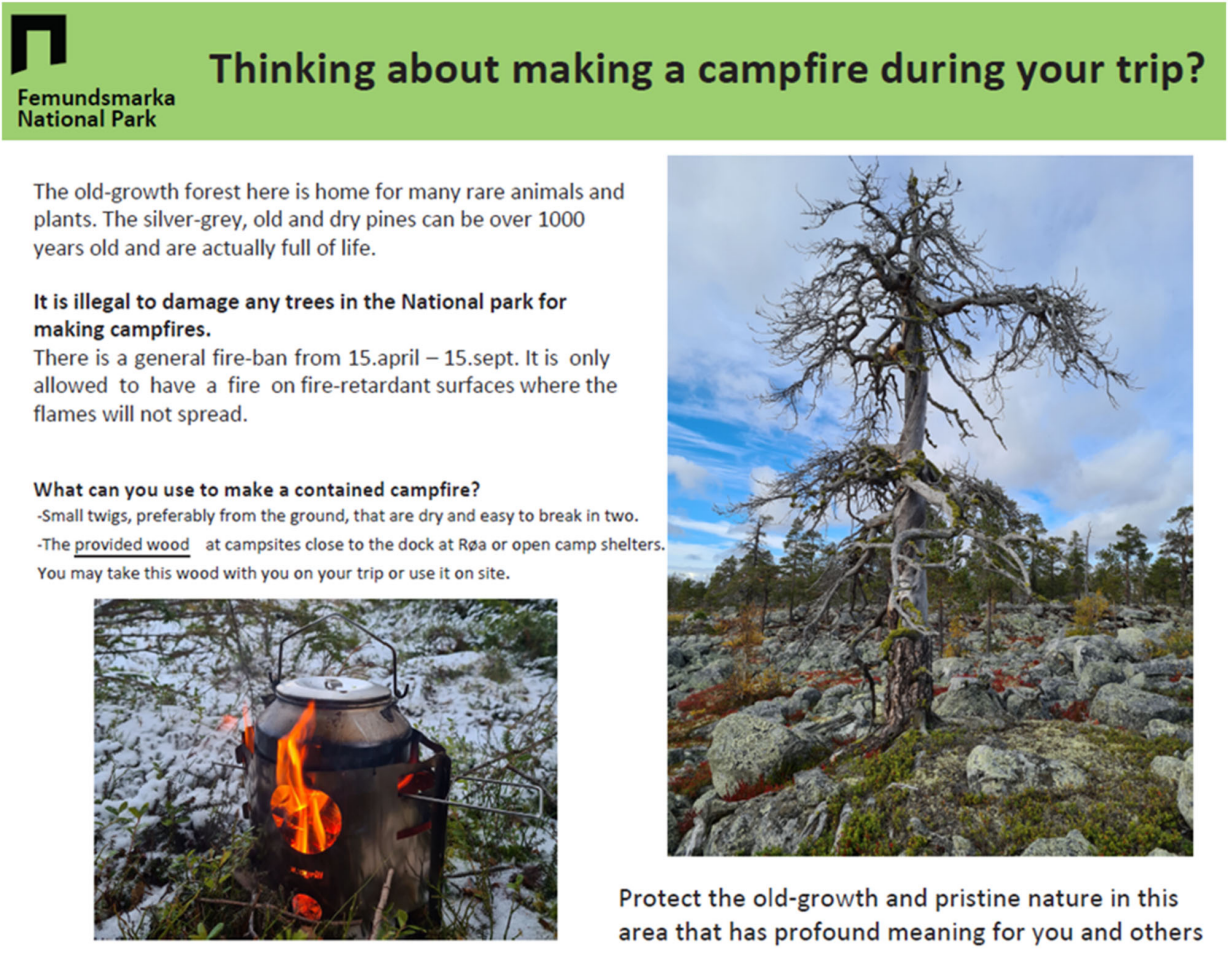
Pamphlet used in the study to change campfire behavior in Femundsmarka National Park, Norway

Since most visitors arrive at Røsanden by ferry, a pamphlet with information was distributed to passengers during the first test period (Fig. 2). In the second test period, the same message was delivered through a brief presentation by a national park employee as visitors disembarked from the ferry. The study was conducted between June 22 and July 29, 2023, and comprised a control period with no specific communication and two experimental periods featuring the different communication methods. The control period initially spanned 13 days. However, unusually warm and dry conditions in early June 2023 heightened the risk of forest fires, leading to an increased focus on fire prevention. Consequently, the abnormal conditions on the first days of the control period influenced studied behavior, and only the last eight days (June 22–29) were utilized as a “control” in the analyses (since the weather changed and regional fire restrictions eased; at the study site, campfires were discouraged rather than explicitly illegal). Each experimental period lasted for 13 days. Between each period, there was a two-day break (June 30 to July 1 and July 15–16) that was implemented to minimize spillover effects and ensure that visitors in receipt of different communication (none, passive, active) were not influenced by each other.

The pamphlet titled *“Thinking about making a campfire?”* was designed to immediately capture attention and clearly signal its purpose (see Fig. 2). It emphasized the potential harm that campfires can cause to trees and biodiversity and featured an image of an old pine native to the area to evoke recognition and concern. The text began by clarifying what is illegal, ensuring visitors understood the regulations, and then offered clear, step-by-step guidance under the question *“What can you use to make a contained campfire?”* To support visitors uncertain about alternatives, this section included a description of where to find provided firewood and an image of a portable stove to illustrate responsible behavior. The pamphlet was crafted in line with recommendations from persuasive communications research, which highlights the importance of designing messages that not only inform but also guide visitors toward feasible alternatives. In particular, encouragement-based prescriptive messages have been shown to be more effective than discouragement-based proscriptive messages, which traditionally dominate outdoor signage (Abrams et al. 2020).

The design of the pamphlet was informed by interviews with visitors during the summer of 2022 and research showing that visitors’ behaviors often align with their self-interests (Abrams et al. 2020; Schultz 2001). The communication concluded with an emotional appeal, aimed at leaving visitors with a lasting impression of their role in protecting Femundsmarka and its wilderness, something that the interviewees showed was important for the visitors. The pamphlet was presented in both Norwegian and English. Additionally, a different test period involved active communication by a national park employee, conveying the same message while also allowing for questions. Upon arrival, all visitors were gathered together at the ferry landing and given a brief talk by one ranger before continuing further into the area. The talk lasted about one minute and followed the same core script as the pamphlet, though the ranger could add a few extra words to adapt the content to the oral format.

### Mapping Tree Damage and Visitor Use

**Fig. 3:**
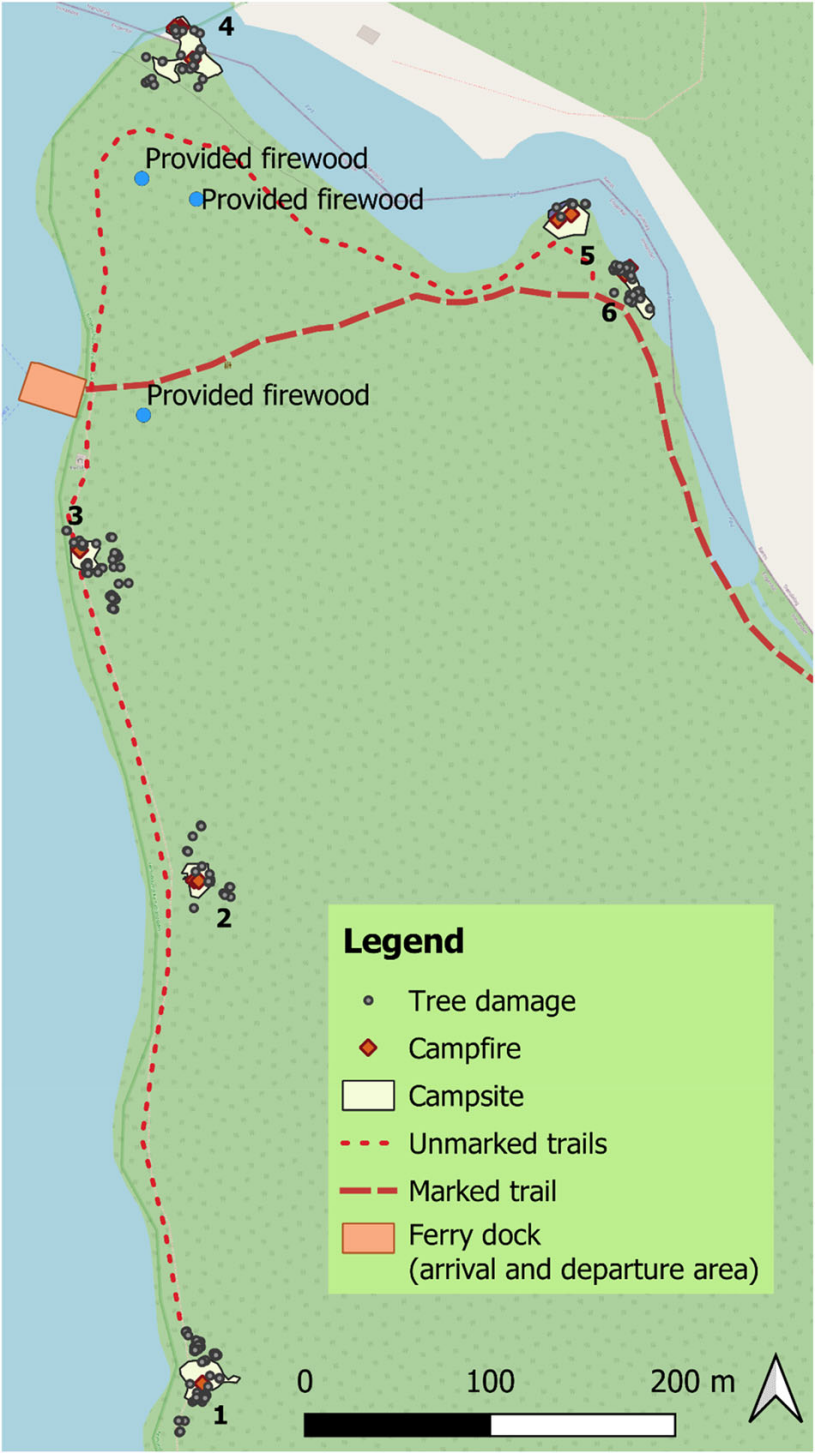
All tree damage incidents recorded at selected campsites during the summer season 2023 at Røsanden, Femundsmarka National Park. QGIS Development Team (2023). QGIS Geographic Information System

To map tree damage, we selected the six most used campsites at Røsanden (Fig. 3). We developed a systematic protocol to record tree damage incidents at each campsite. Before the start of the study, on June 16, 2023, all tree damage incidents were recorded and categorized by type and age. We used tablets with ArcGIS FieldMaps (Pro version 3.1) to collect photos, drawings, GPS positions, and detailed descriptions, to avoid double-counting the same damage. Subsequently, we revisited the selected campsites every second day during the study period and systematically recorded new instances of tree damage (protocols available upon request to authors). The damage incidents were categorized as wound, ripped bark, broken twig, cut branch, or whole tree. The specified radius around the investigated campsites was 50 meters, starting from where the vegetation cover was intact, thereby delineating a boundary for each campsite (Fig. 3). The selected radius was demarcated based on where most damage incidents had been observed in the pretest in 2022. The visitor numbers were based on manual counts of people at the selected campsites, recorded every other morning before campers took down their tents and left the sites, and before tree damage was surveyed.

To map visitor characteristics and behavior, we distributed on-site questionnaires to visitors who had camped overnight at Røsanden (*n* = 149, 97% response rate). The aim of the survey was to assess whether visitors in different test periods differed in terms of characteristics that might have affected their campfire behavior. The survey collected visitors’ general demographic information, including age, gender, nationality, experience of the area and outdoor activities in general, details about their visit such as duration and primary motivations, and their perceptions regarding campfires and firewood collection behavior in the area. Because the visitor survey was conducted as visitors were about to leave and the mapping of tree damage was carried out afterward, neither activities influenced campfire behavior during the stay.

### Data Treatment and Analysis

As far as the authors are aware, the behavior surrounding campfires and the use of firewood has not been studied in this manner before. Consequently, the selection of independent variables in statistical analysis was not grounded in existing theories or literature. Instead, it was based on field observations and visitor interviews conducted in 2022 (see Online Resource 2). Nonetheless, our choice of predictors is conceptually grounded in prior work on depreciative behaviors, often arising from limited skill or information (Marion and Reid 2007). Factors believed to influence tree damage included self-reported campfire perceptions and behavior, outdoor experience, prior experience of Femundsmarka, weather conditions, visitor number, and our passive/active communication treatment.

We recorded tree damage and campsite visitor numbers every second day, based on the typical two-day visitor stay, and standardized them to daily values by dividing by two for analysis. Survey data were averaged across the same two-day intervals. The survey highlighted factors affecting campfire behavior, including responsible firewood use, opting out of campfires, and avoiding tools like axes or saws, patterns that were also reported in visitor interviews (2022; see Online Resource 2). We aggregated campfire-related responses (Online Resource 1) into a “self-reported campfire behavior” score, with higher values showing responsible practices. This score was included as a covariate in the statistical analyses, as a proximal indicator of campfire-related knowledge and attitudes. Self-reports can be biased but are often informative about related practices (Christensen and Cole 2000), which were consistent with themes from the visitor interviews and observations. Additionally, we assumed that experienced outdoor visitors and those familiar with Femundsmarka might prioritize campfires and firewood collection as integral parts of their experience, as indicated in visitor interviews. Greater skill and site-specific knowledge (Marion and Reid 2007) could also mitigate uninformed practices such as cutting live branches. We included visitor numbers to represent use-level pressure, given well-established links between visitation and environmental impacts (Manning 2017). Daily weather conditions (“wind speed,” “rainfall,” “temperature”) were obtained from the nearest official meteorological station at Drevsjø (Yr 2023). Weather was included in the analyses, as we anticipated it would impact campfire behavior: fewer fires were expected during rainy or windy conditions, with temperature’s impact uncertain, colder days might encourage more fires for warmth or discourage activity altogether. This expectation was echoed in visitor interviews and supported by field observations (fewer active fire rings on wet/windy days).

Analyses were conducted in R (v.4.2.3, R Core Team 2023), using mainly the base library. To examine visitor differences across test periods, we used one-way ANOVA with Tukey HSD post hoc tests for normally distributed data, and Kruskal-Wallis tests with Dunn-Bonferroni corrections for non-normal data. Categorical data were analyzed with chi-square tests and Bonferroni correction. The unit of analysis was per respondent for testing differences across test periods (*N* = 149), and per day for examining tree damage levels (*N* = 34). We employed linear regression to examine factors affecting campfire behavior, using number of tree damage incidents as the outcome variable (averaged bi-daily across campsites). Initial models included test period, self-reported campfire behavior, outdoor experience, prior experience of the area, visitor number, and weather variables (temperature, wind, rainfall). A stepwise model selection with Akaike information criterion (AIC) was used to find the most parsimonious model, with a significance threshold of α = 0.05 (Burnham and Anderson 2002), using the AICcmodavg library in R (Mazerolle 2023). Interaction terms (e.g., between weather and treatment) were tested but did not improve model performance. Post hoc comparisons between control, passive, and active communication periods were conducted with Tukey tests. For the final model, we collected unstandardized regression coefficients (B, “Estimate”), standardized coefficients (β), and partial η² for each predictor to describe both the direction/strength of relationships and their unique contribution to variance in tree damage, using lm.beta (Behrendt 2025). We verified model assumptions with q-q plots for normality and homoscedasticity of residuals. We analyzed differences in damage types across treatment periods using Kruskal-Wallis tests, the outcome was the count of occurrences of each damage type and the predictor was treatment. Post hoc comparisons between the test periods were performed using Dunn’s test with Bonferroni corrections. For general descriptions of these statistical methods and procedures, see e.g. Zar (2010).

## Results

### Characteristics of the Test Periods

Across the three test periods in the summer of 2023 (22–29 June, 2–14 July, and 17–29 July), visitors exhibited similar characteristics overall (Online Resource 1), with some small variations: visitors in the first period (control) tended to be older compared particularly to those in the third period (active communication). Furthermore, a slightly higher proportion of visitors were foreigners in the second period (passive communication), 35% compared with about 16% in the other periods. Importantly, visitors expressed similar opinions regarding campfire behavior and tree damage incidents across all three time periods (no statistically significant differences; Online Resource 1). The three explanatory variables, “Self-reported campfire behavior,” “Outdoor recreation experience,” and “Prior experience of Femundsmarka” showed no variation among visitors across the three test periods, meaning there were no systematic differences in visitor characteristics that potentially would override the effects of the communication.

**Fig. 4:**
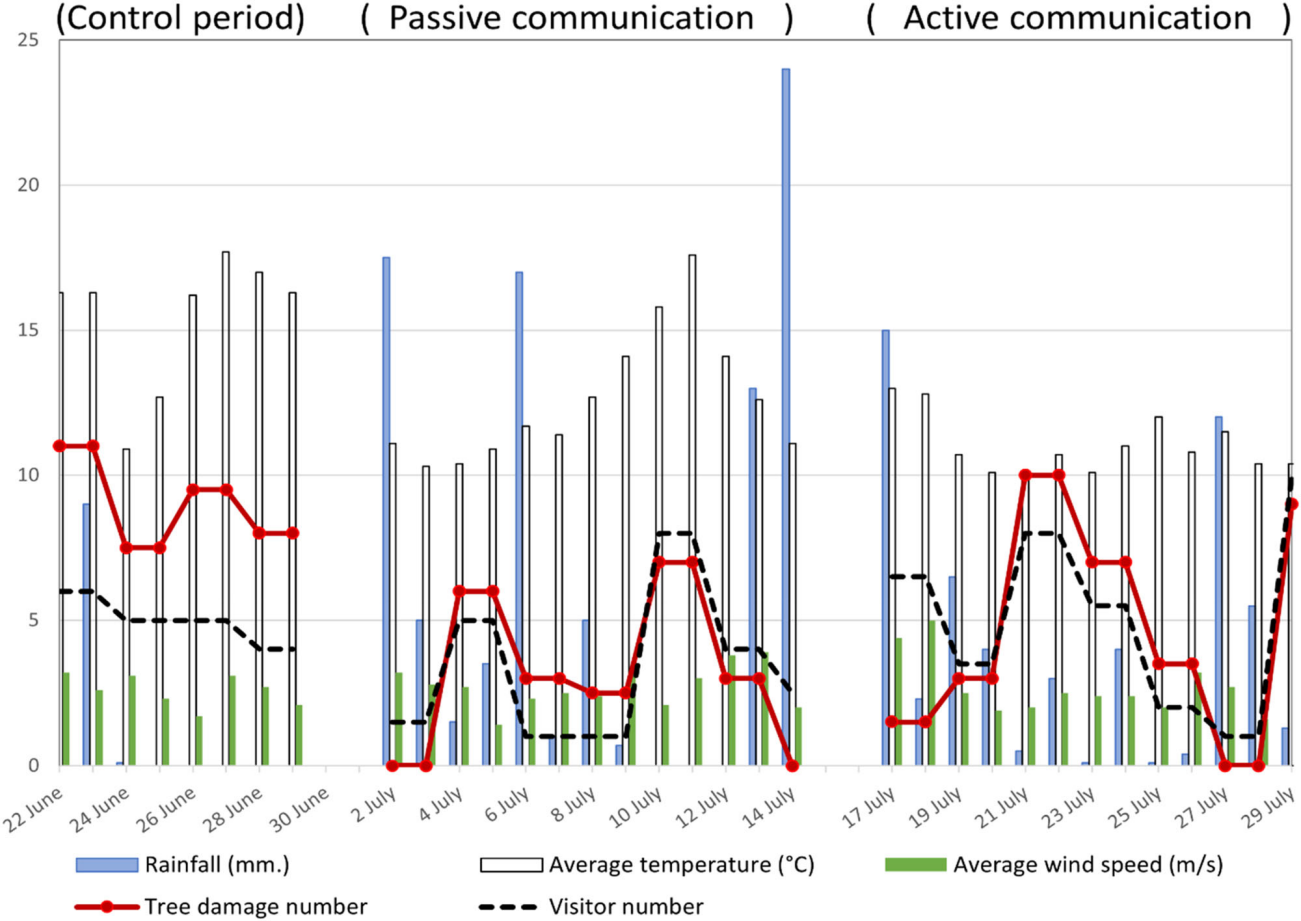
The weather conditions (recorded daily), visitor number and tree damage numbers (recorded bi-daily) at six selected campsites at Røsanden, Femundsmarka National Park, during the summer season 2023. The y-axis represents all variables, and the unit of measurement for each variable is given in the legend. Created in Microsoft Corporation. (2024). *Microsoft Excel* (Version 16.0)

We observed a decline in the number of visitors camping at Røsanden during the passive communication phase (daily average of three people, 2–14 July, compared to five people in the other two periods; Fig. 4), but this difference was not statistically significant. Weather data showed a shift in conditions from June to July, characterized by colder temperatures and more rain and wind (Fig. 4). The mean temperature was significantly higher and mean daily rainfall significantly lower in the control compared to in the two treatment periods, while wind conditions did not vary significantly. Visitor numbers and tree damage were obtained from manual counts at the selected campsites every other day, with daily values estimated by dividing these counts by two, whereas weather data were available for every day from the meteorological station.

### The impact of passive and active communication on tree damage

**Fig. 5:**
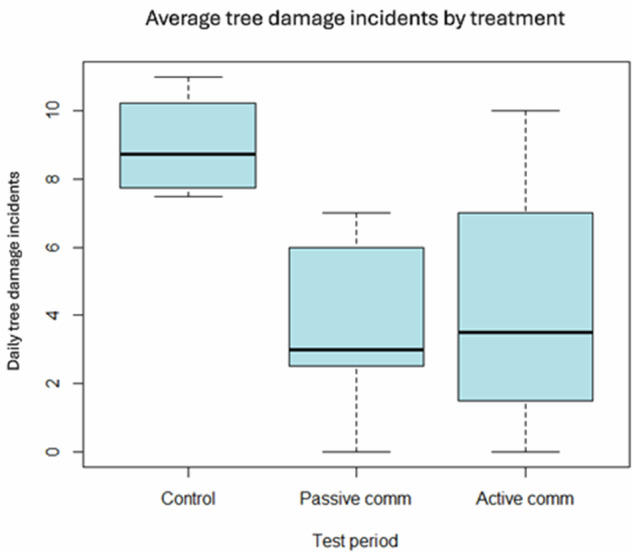
Box and whisker plot showing average daily tree damage incidents by treatment. The thick line shows the median and the box covers the interquartile interval, where 50% of the data occurs. The whiskers extend from the minimum to the lower quartile and from the upper quartile to the maximum

Between June 22 and July 29, 2023, we documented 174 tree damage incidents across the six designated campsites, dominated by ripped bark and broken twigs (Table 1). The daily occurrence of tree damage incidents varied from 0 to 11, with on average 9.0 per day during the control period, 3.3 per day during treatment period 1 (passive communication), and 4.5 during treatment period 2 (active communication) (Fig. 5). Using a model with test period as the only explanatory variable, we found that the mean daily number of damage incidents was significantly lower in both treatment periods than in the control period (F = 10.41, *p* < 0.001, adjusted R^2^ = 0.36). On average, based on the recorded number of tree damage incidents (Table 1), campsites experienced a 57% reduction in damage when the communication interventions were implemented compared to when they were not. We also found fewer cut branches in the treatment periods than in the control period.

**Table 1:** Number of tree damage incidents per damage type and mean daily damage in each test period. Illegal tree damage in Femundsmarka National Park is limited to cut branches and whole trees

Test period	Wound	Ripped bark	Broken twig	Cut branch	Cut whole tree	Days	Average number of daily damage incidents
**Period 1 (Control)**	0	26	35	11	0	8	9.0
**Period 2 (Passive)**	2	21	17	3	0	13	3.3
**Period 3 (Active)**	5	30	21	3	0	13	4.5

The best model to explain tree damage numbers confirmed that mean daily number of damage incidents at the six campsites was lower in the two treatment periods compared to in the control period (Table 2). Furthermore, the number of tree damage incidents increased with the number of visitors, but it was lower on days with more rain or wind, although their standardized effects were smaller than those of treatment and visitor numbers. Both the treatment factor (partial η² = 0.73) and visitor numbers (partial η² = 0.70) explained a substantial proportion of the variance in tree damage (Table 2). The model seemed to be a good fit and there was no indication of non-linearity, heteroscedasticity, or substantial deviation from normality (see Online Resource 3). The model explained 82.5% of variation in the number of tree damage incidents (F = 32.10, *p* < 0.001, adjusted R^2^ = 0.825). In comparison, a model using only test period explained 36% of the variation. There was no significant difference in tree damage incidents between treatment period 1 or 2 (*p* = 0.52, Tukey post hoc test). The standardized coefficients in the main model also suggest that both communication treatments had similarly large effects on tree damage incidents, with active communication showing a slightly larger reduction than passive communication. The variables self-reported campfire behavior, experience of outdoor recreation, prior experience of Femundsmarka, and daily temperatures did not contribute significantly to explaining variation in tree damage (see Online Resource 3).

**Table 2:** Model parameters from the optimal linear model explaining tree damage incidents at six campsites at Røsanden, Femundsmarka National Park, in June and July 2023, from a linear regression model

	Estimate	Standard error	t-value	*p* value	β (std)	Partial n^2^
Intercept	7.734	1.122	6.894	<0.001	–	–
Passive communication	–3.546	0.723	–4.904	<0.001	–0.49	0.73
Active communication	–3.927	0.682	–5.757	<0.001	–0.54	0.73
Visitor number	0.841	0.114	7.357	<0.001	0.59	0.70
Wind speed	–1.080	0.335	–3.223	0.003	–0.24	0.31
Rainfall	–0.113	0.046	–2.456	0.021	–0.20	0.18

When considering tree damage types, counts in the different categories were generally small, and the models had predominantly limited explanatory power. However, we found significant differences in illegal damage incidents (meaning cut branches, no cut trees were found during the study) (H(2) = 11.42, *p* = 0.003), indicating fewer incidents during treatment periods compared to in the control period. A Dunn–Bonferroni post hoc comparison showed no difference between the two treatment periods (*p* = 1.00). During the control period, an average of 1.4 illegal tree damage incidents occurred each day, while both treatment periods experienced on average a 0.23 illegal tree damage incidents per day.

## Discussion

We asked whether communication reduces tree damage, expecting lower damage during periods when visitors received information, especially when it was delivered face-to-face. In brief, communication reduced tree damage, but spoken delivery was not more influential in changing behavior than passive delivery. Our aim was also to understand how communication operates under real-world conditions, accounting for visitor volume, visitor characteristics, and weather. We observed more tree damage on days with higher visitor numbers, and less damage on windy or rainy days. Taken together, the findings suggest that well-designed communication can meaningfully influence campfire behavior, while weather, and, to a lesser extent, visitor characteristics, also shape behavior. These patterns are consistent with intention–behavior gaps and other contextual factors that moderate intervention effects in the field (Ardoin et al. 2015; He et al. 2023). Our findings show that communication strategies, when designed and delivered in line with behavioral and communication theory, can reduce damaging practices while maintaining positive visitor experiences. However, environmental conditions must be considered alongside communication, as they also explain visitor behavior and can modulate the effectiveness of interventions.

### Passive and Active Communication Strategies

Our results supported the first hypothesis: communication reduces tree damage, with lower levels of damage during periods when visitors received information compared to control periods without communication. This pattern is consistent with previous research showing that harmful recreation behavior often stems from a combination of uninformed or unskilled actions, inconsistent regulations, and habitual routines (Marion and Reid 2007). Theories of behavior change help explain these patterns: the Theory of Planned Behavior (Ajzen and Fishbein 1980) and Value-Belief-Norm theory (Stern 2000) highlight how values, attitudes, perceived opportunity, and responsibility shape behavioral intentions. Analysis of visitor interviews showed that campfire behavior worked through these very mechanisms, revealing widespread uncertainty about campfire regulations and a strong attachment to campfires as part of the nature experience, alongside a desire to protect trees. The solution therefore, aimed to make campfires possible without damaging trees, and the COM-B model (Michie et al. 2014) clarified how the communication supported this goal by enhancing capability (clarifying what was legal and which tools to use), opportunity (pointing visitors toward provided firewood), and motivation (by appealing to a sense of responsibility for protecting ancient pines). By tailoring the message communication proved effective consistent with Hypothesis 1. While both interventions reduced damaging practices, neither eliminated them entirely, underscoring the difficulty of changing behaviors that are habitual and culturally embedded (Serenari et al. 2012; Stern 2000).

Our second hypothesis: active communication, a short face-to-face message delivered by a national park employee, is more effective, meaning it reduces tree damage more than passive communication through pamphlets, was only partly supported. Illegal tree damage occurred less frequently when communication was provided than during the control period, and minor tree damage also decreased. Focusing only on tree damage incidents, illegal damage occurred with similar frequency in the two communication periods, whereas minor tree damage was actually more frequent during active communication than during passive communication (Table 1). A likely reason is that the pamphlet sometimes left visitors uncertain about what was actually permitted, leading several to refrain from using wood and twigs from ancient trees altogether. In contrast, active communication provided direct engagement and opportunities to ask follow-up questions, giving visitors complete certainty about what was allowed, but it also clarified that some wood collection was legal, which paradoxically made minor tree damage feel more acceptable to some visitors. These patterns support earlier concerns that inconsistent regulations and mixed messages from land managers can complicate efforts to promote responsible behavior (Aas et al. 2022; Stubbs 1991).

While these descriptive patterns highlight how visitors responded to the two communication formats, they also connect to a key aim of this study: to understand how such interventions operate under real-world conditions and capture the complexity of natural settings, where ecological factors and situational dynamics interact. If we look at the linear regression model that accounts for weather and visitor volume/characteristics, active communication can be interpreted as slightly more effective than passive communication. This also highlights that the full model explains a much larger proportion of the variation in tree damage incidents than analyses that only compare communication conditions. Together, these points underline the importance of evaluating communication strategies under realistic field conditions, where factors such as visitor volume, visitor profiles, and weather are included.

Taken together, these results indicate that, although the estimated effects of passive and active communication differ slightly depending on the level of adjustment, the differences are small, and both strategies ultimately reduce tree damage incidents by roughly half compared to days without communication. In our data, the pattern fits behavioral/norm-based theory best. Guided by COM-B, the interventions were effective because both formats addressed capability, opportunity and motivation, which aligns with TPB/VBN. This study also supports research showing that nature-based tourists generally value educational experiences and are receptive to environmental information (Ardoin et al. 2015). At the same time, communication theory stresses the importance of design, delivery, and context (Petty and Cacioppo, 1986). We did not find a clear advantage of face-to-face over passive delivery, suggesting that what we communicated and the practical support mattered more than the channel. In our case passive communication via pamphlets worked effectively, likely because the written material offered a good opportunity for engagement on the ferry, where visitors had time and most chose to read it, and because it could be revisited later, reinforcing descriptive and injunctive norms over time.

Despite these communication efforts and the provision of designated firewood, tree damage persisted to some extent. Our study revealed that campfires hold significant value in providing positive experiences for many visitors to Femundsmarka. Interview analyses showed that visitors view campfires as integral to ‘the wilderness feeling’ linked to tradition, social bonding, comfort, the enjoyment of collecting firewood, and a sense of freedom (Online Resource 2). Building campfires and collecting firewood are integral parts of the experience, offering a sense of seclusion from the wider crowd, environmental mastery, and social interaction (Christensen and Cole 2000; Lucas 1980).

Overall, these results highlight that communication effectiveness is not a fixed outcome but depends on how content is framed and delivered, how it interacts with visitors’ existing beliefs, and the prevailing environmental conditions (D’Antonio et al. 2012; Guo et al. 2015; Schwartz et al. 2018).

### Broader Implications for Management

Tourism and outdoor recreation are significant drivers of environmental degradation (Monz et al. 2010), reinforcing the urgency for integrated management strategies that directly address visitor behavior (Guo et al. 2015). Our findings contribute to this agenda by showing that relatively simple communication measures, when grounded in behavioral and communication theory, can meaningfully reduce ecological impacts without undermining the visitor experience. In our case, active communication took the form of brief group announcements on the ferry dock rather than one-on-one conversations with each party, which likely limited the opportunity to tailor messages. In addition, distractions such as children, dogs, heavy backpacks, and the practicalities of boarding and disembarking may have reduced how fully visitors engaged with the message, which may help explain why active communication was only slightly more effective than passive communication, despite the potential advantages of direct interaction.

Beyond communication, we recommend that park management increase monitoring at high-use campfire areas by conducting routine checks of tree condition, visitor practices, and firewood use, reinforcing rules on site and coordinating timely responses (e.g., replenishing designated firewood). Given that some illegal tree damage persisted even during communication periods, these efforts should be complemented by clear legal regulations and the possibility of sanctions (e.g., warnings or fines) in line with national park rules. Given limited personnel capacity, this may require seasonal staffing (e.g., a dedicated ranger) or partnerships with volunteers and local clubs. At the same time, the study revealed that environmental context, particularly weather, shaped outcomes: periods of strong wind and heavy rain – not surprisingly – discouraged campfire activity, although being of less importance than visitor numbers. These patterns underscore that actual behavior is influenced not only by communication but also by dynamic natural conditions (Guo et al. 2015). Moreover, self-reported campfire behavior did not predict actual practices, supporting the well-documented behavior–intention gap (Ardoin et al. 2015; He et al. 2023). This underscores the importance of investigating observed behavior in combination with the dynamic and changing conditions at a site.

Although other studies suggest that less-experienced recreationists may be more willing to follow rules because they lack entrenched habits (Selvaag and Evju 2025), neither general outdoor recreation experience nor prior visits to Femundsmarka significantly influenced campfire behavior in our case. This indicates that the drivers of campfire practices lie less in individual experience and more in shared cultural traditions. Campfires carry deep symbolic meanings of freedom, self-reliance, and sociality (Christensen and Cole 2000, Hammitt et al. 2015), which cannot be addressed by regulations alone. Indeed, strict bans may be both impractical due to access rights (Fredman et al. 2021) and undesirable because they risk undermining valued experiences or displacing visitors (Gundersen et al. 2015). Instead, behavioral oriented strategies that combine communication with supportive infrastructure appear more promising, as our data shows that daily tree damage incidents were reduced by 57% on days with communication compared to days without communication. For example, our findings align with evidence from Switzerland showing that providing firewood and designated fireplaces reduces ecological damage while accommodating visitor preferences (Hegetschweiler et al. 2009).

Our results highlight the importance of place-specific solutions, echoing the argument that environmental communication must be rooted in local traditions, norms, and management contexts (Ardoin et al. 2015; Serenari et al. 2012). What works in Femundsmarka may not work elsewhere, but the broader principle remains: behavior-oriented approaches that combine communication, social norms, and supportive infrastructure can reduce impacts while sustaining meaningful visitor experiences. In this sense, visitor communication should be seen not merely as information transfer but as part of a broader care-oriented approach to environmental management, fostering relationships between people, place, and ecosystems (Brooks and Champ 2006).

### Strengths and Limitations of the Study

Our quasi-experimental field study purposely considered contextual and site-specific human and non-human factors, and it utilized multiple methods and disciplines. It was not possible to monitor each visitor at every campsite continuously in the field; therefore, instead of directly correlating the visitors’ actual behavior with their questionnaire responses, we aggregated visitor data across all sites. Attempting to link individual behavior to survey responses would have risked making participants aware of the experiment, potentially altering their actions independently of the communication interventions. Because we recorded tree damage incidents and visitor numbers only every other day, variation in damage due to factors such as weather conditions may be masked in the dataset. Nevertheless, our analyses showed that fewer damage incidents occurred on windy and rainy days. Internal validity (i.e., certainty that changes in the manipulated variable caused changes in the dependent variable) might be lower in our study than in a replicated experimental study. However, designing such experiments within this field of research would face other challenges, primarily that the experiments might not be realistic or would be too complex and costly.

We designed the study to minimize confounding elements by maintaining consistent circumstances and matching other factors, and we found no systematic differences in visitor characteristics across test periods. Nevertheless, achieving complete confidence that confounding factors is not at play is challenging. Sample size combined with effect sizes plays a crucial role in Type I errors. However, due to practical constraints limiting our data collection to six weeks, with a shortened control period due to forest fire caution, our sample size was restricted. In conclusion, while individual data points warrant caution regarding statistical rigor, the overarching pattern of findings remains less vulnerable to Type I errors. The resultant pattern persisted across our analytical methods: Communication impacted visitors and led to less tree damage. The findings provide actionable insights for researchers and managers seeking to design and evaluate communication-based interventions, and they underscore the value of field experiments as a foundation for future research on how visitors respond to environmental communication strategies.

## Supplementary information

Supplementary information

## Data Availability

Data is provided within the manuscript or supplementary information files and additional datasets of this study are available from the Norwegian Institute for Nature Research (NINA), but restrictions apply to the availability of these data, which were used under license for the current study and so are not publicly available. The data are, however, available from the authors upon reasonable request and with the permission of the Norwegian Agency for Shared Services in Education and Research (SIKT).
