# Supplementary material for: Fostering Pro-environmental Behavior Among National Park Visitors: Testing Communication Strategies for Campfire Management
Source: Environ Manage. 2026 Feb 18;76(3):99. doi: 10.1007/s00267-026-02392-6 (PMC12916532; doi:10.1007/s00267-026-02392-6)
Supplement: Supplementary file 1 — Supplementary information [file 267_2026_2392_MOESM1_ESM.docx]

**Online Resource 1. Visitor characteristics**

**Table 3**. Visitor demographics, visit characteristics, outdoor recreation, and experience of Femundsmarka: percentage distribution across test periods. Rows with variables shown in bold font were tested statistically and show ± standard deviations, whereas other rows show the distribution of the variables tested. Age, gender, and nationality were assessed to explore their potential influence on tree damage levels.

|  | Control  (N =40) | Passive communication 2 (N=46) | Active communication 3 (N=63) |
| --- | --- | --- | --- |
| **Mean Age-** | **42 yrs±17*** | **41 yrs ± 12** | **35 yrs ± 13*** |
| Age 18-30 | 35% | 24% | 50% |
| Age 31-50 | 28% | 52% | 32% |
| Age < 50 | 38% | 24% | 18% |
| **Mean trip duration-** | **4 days ± 1.8** | **5 days ±1.9** | **5 days ± 2.9** |
| Trip between 1-3 days | 35% | 35% | 35% |
| Trip between 4-7 days | 63% | 59% | 48% |
| Trip longer than 7 days | 3% | 7% | 17% |
| **Mean outdoor recreation experience (scale 1–6)”** | **4.7 ± 1.5** | **4.9 ± 1.4** | **4.5 ± 1.5** |
| Unexperienced with multi-day hikes (< 6 times, 1-3) | 20% | 24% | 25% |
| Mid experienced with multi-days hikes (6-20 times, 4-5) | 35% | 20% | 40% |
| Very experienced with multi-day hikes (more than 20 times, 6) | 45% | 56% | 35% |
| **Mean prior experience of Femundsmarka (scale 1–7)”** | **2.8 ± 2.3** | **2.5 ± 2.2** | **2.3 ± 2.0** |
| First-time visitor to Femundsmarka | 50% | 59% | 59% |
| Visited Femundsmarka 2-4 times | 28% | 24% | 27% |
| Visited Femundsmarka more than 4 times | 22% | 17% | 14% |
| **Male^** | **85%** | **74%** | **70%** |
| **Norwegian^** | **82%** | **65%*** | **86%*** |

* We detected a statistically significant difference in means (p < 0.05) between those groups through a one-way ANOVA, followed by a Tukey HSD post hoc test (-), Kruskal-Wallis, followed by either a Dunn-Bonferroni post-hoc test (“) or a chi-square test of independence followed by Bonferroni correction (^).

**Table 4**. The respondents were presented with a series of statements related to campfires and tree damage, and they were asked to assess each statement’s alignment with their behavior during a trip to Femundsmarka National Park. They rated each statement on a scale ranging from 1 (strongly disagree) to 7 (strongly agree). Below are the averages for respondents from each test period and ± standard deviations. We detected no statistically significant difference in means (p < 0.05) between the specific groups through non-parametric Kruskal-Wallis tests.

|  | Control (N =40) | Passive communication (N = 46) | Active communication (N = 63) |
| --- | --- | --- | --- |
| I usually take an axe and/or saw with me | 3.1 ± 2.3 | 3.3 ± 2.1 | 2.6 ± 2.2 |
| Stumps and dead trees are important for biodiversity | 6.2 ± 1.2 | 6.1 ± 1.3 | 6.1 ± 1.2 |
| I only use twigs I find on the ground for campfires | 5.2 ± 2.1 | 5.1 ± 2.2 | 4.9 ± 2.1 |
| It bothers me to see a tree cut down by others | 5.2 ± 1.9 | 5.5 ± 1.7 | 5.3 ± 1.9 |
| I only use the commercial wood at maintained campsites for making a campfire | 4.1 ± 2.1 | 4.4 ± 1.2 | 4.2 ± 2.2 |
| I have seen too much damage to trees caused by other visitors | 3.5 ± 2.0 | 3.3 ± 1.8 | 3.4 ± 1.9 |
| I always avoid making a campfire | 2.3 ± 1.8 | 2.9 ± 1.8 | 2.6 ± 2.0 |
| **Self-reported campfire behavior^** | **4.5 ± 0.9** | **4.6 ± 0.9** | **4.6 ± 0.9** |

^ The seven campfire-related factors were combined into the variable “self-reported campfire behavior”. Responses to the statement, “I usually take an axe and/or saw with me”, were reversed. Higher values in “self-reported campfire behavior” indicated more responsible campfire conduct.

**Online Resource 2. Pre-study 2022: Components influencing campfire behavior at Røsanden**

To gain insight into campfire behavior among visitors at Røsanden, a combination of coupled semi-structured interviews (held with visitors, n = 11) and field observations (n = 22) were conducted in 2022. The findings were analyzed, and the COM-B model (Michie et al. 2014, see References for details) was employed to categorize and structure the findings into three distinct bundles.

Many visitors expressed that they did not use a campfire to cook food, as they had portable stoves for that purpose, but many used campfires to dry clothes, create a cozy and warm atmosphere, and/or keep mosquitoes away. The motivation (M) to exhibit the desired behavior was therefore less because campfires were important for many visitors, and it was socially accepted and important for a wilderness experience. At the same time, ancient pines and pristine nature were a significant part of the nature experience, and keeping the nature pristine was important to most of the interviewed visitors. The national park status also motivated interviewed visitors to protect the environment and behave differently than they would in non-protected areas.

The scheme with firewood provided for visitor use at designated sites was warmly received by the visitors, yet it remained unknown to all interviewees that it was available for them to use. While visitors appeared to have the opportunity (O) to engage in the desired behavior, such as utilizing alternative cooking equipment or the provided designated firewood, their lack of awareness regarding firewood options and the significance of preserving trees was evident. Additionally, a common practice observed was that the wood from ancient trees at campsites was left for the next visitors, establishing it as an accepted social norm. Based on the results from the COM-B analyses, the message design was crafted in collaboration with a communication advisor, graphic designer, national park managers, and a reference group with experience in similar projects.


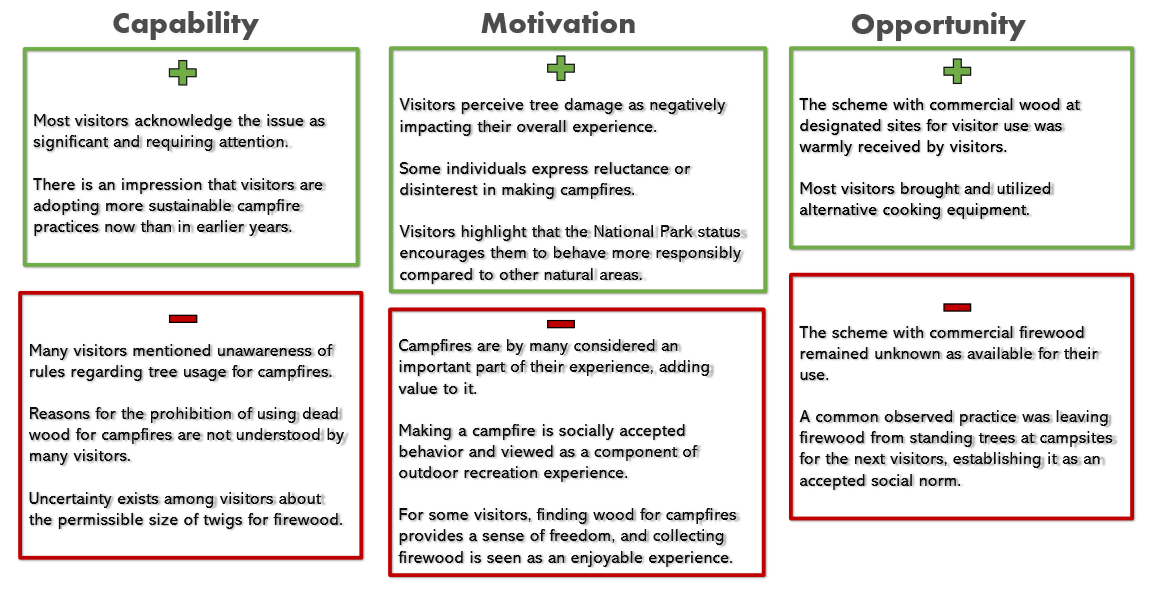


**Fig. 7** Components in green boxes with plus signs are regarded as positive factors for shifting campfire behavior towards a more sustainable direction, whereas components in red boxes with minus signs are seen as obstacles to reducing tree damages by visitors

**Online Resource 3. Detailed description of analyses and results of tree damage models**

To select the most parsimonious model to explain tree damage number, we used AIC and backward elimination to explore alternative models, with a significance threshold of α=0.05 (Table E1).

**Table 5**. Model selection results. The table shows AIC values (AICc), difference in AICc-values from the best model (Delta_AICc), the proportion of the predictive power of the complete dataset (AICcWt), and the cumulative predictive power (Cum.Wt). In the model column, we show the explanatory variables included in the model.

| Model | AICc | Delta_AICc | AICcWt | Cum.Wt |
| --- | --- | --- | --- | --- |
| Test period+Visitor number+Wind speed+Rainfall | 135.20 | 0.00 | 0.61 | 0.61 |
| Test period+Visitor number+Wind speed+Rainfall+ Outdoor recreation experience | 137.43 | 2.23 | 0.20 | 0.82 |
| Test period+Visitor number+Wind speed | 138.63 | 3.44 | 0.11 | 0.93 |
| Test period+Visitor number+Wind speed+Rainfall+ Outdoor recreation experience+ Self-reported campfire behavior | 140.71 | 5.51 | 0.04 | 0.97 |
| Test period+Visitor number+Wind speed+Rainfall+ Outdoor recreation experience+ Self-reported campfire behavior+ Prior experience of Femundsmarka | 141.29 | 6.09 | 0.03 | 0.99 |
| Test period+Visitor number+Wind speed+Rainfall+ Outdoor recreation experience+ Self-reported campfire behavior+ Prior experience of Femundsmarka+Temperature | 145.61 | 10.42 | 0.00 | 1.00 |
| Test period+Visitor number | 146.38 | 11.18 | 0.00 | 1.00 |
| Test period | 173.63 | 38.44 | 0.00 | 1.00 |

The full model including all seven explanatory variables had poor fit, and outdoor recreation experience, self-reported campfire behavior, prior experience of Femundsmarka and temperature were non-significant. We thus tested different simpler models. The best model included test period, visitor number, wind speed and rainfall as explanatory variables, containing 61 % of the total explanation that could be found in the full set of models.


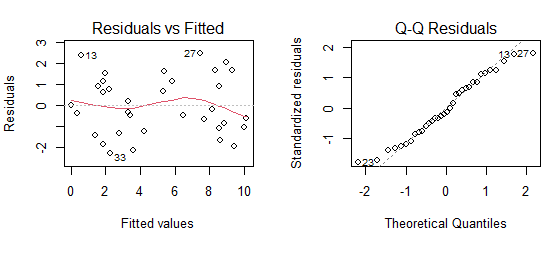


**Fig. 8** The diagnostic plots for the linear regression model (Tree damage~Test period+Visitor number+ Wind speed+Rainfall) include scatterplots that illustrate the prediction errors (residuals) plotted against the predicted values. These plots provide a visual assessment of the model's performance and its ability to accurately predict tree damage levels based on the treatment variable (Test period) and visitor number, wind speed and rainfall variables
